# Intraoperative assessment of anastomotic blood supply using indocyanine green fluorescence imaging following esophagojejunostomy or esophagogastrostomy for gastric cancer

**DOI:** 10.3389/fonc.2024.1341900

**Published:** 2024-01-18

**Authors:** Peng Wang, Yantao Tian, Yongxing Du, Yuxin Zhong

**Affiliations:** Department of Pancreatic and Gastric Surgery, National Cancer Center/National Clinical Research Center for Cancer/Cancer Hospital, Chinese Academy of Medical Sciences and Peking Union Medical College, Beijing, China

**Keywords:** indocyanine green, fluorescence imaging, laparoscopic gastrectomy, anastomotic blood supply, anastomotic leakage

## Abstract

**Objective:**

This retrospective study aimed to evaluate the feasibility and safety of intraoperative assessment of anastomotic blood supply in patients undergoing esophagojejunostomy or esophagogastrostomy for gastric cancer using Indocyanine Green Fluorescence Imaging (IGFI).

**Materials and methods:**

From January 2019 to October 2021, we conducted a retrospective analysis of patients who had undergone laparoscopic gastrectomy for the treatment of gastric cancer. The patients were consecutively enrolled and categorized into two study groups: the Indocyanine Green Fluorescence Imaging (IGFI) group consisting of 86 patients, and the control group comprising 92 patients. In the IGFI group, intravenous administration of Indocyanine Green (ICG) was performed, and we utilized a fluorescence camera system to assess anastomotic blood supply both before and after the anastomosis.

**Results:**

The demographic characteristics of patients in both groups were found to be comparable. In the IGFI group, the mean time to observe perfusion fluorescence was 26.3 ± 12.0 seconds post-ICG injection, and six patients needed to select a more proximal resection point due to insufficient fluorescence at their initial site of choice. Notably, the IGFI group exhibited a lower incidence of postoperative anastomotic leakage, with no significant disparities observed in terms of pathological outcomes, postoperative recovery, or other postoperative complication rates when compared to the control group (p > 0.05).

**Conclusion:**

This study underscores the potential of IGFI as a dependable and pragmatic tool for the assessment of anastomotic blood supply following esophagojejunostomy or esophagogastrostomy for gastric cancer. The use of IGFI may potentially reduce the occurrence of postoperative anastomotic leakage.

## 1 Introduction

According to the most recent global cancer burden data, gastric cancer ranks as the fifth most prevalent malignancy and the fourth leading cause of cancer-related mortality, with an overall mortality rate of 7.7 per 100,000 individuals (1). In China, the absence of extensive gastric endoscopy screening programs has led to the predominance of locally advanced gastric cancer cases (2–4). For upper-middle gastric cancer, total or proximal gastrectomy remains the primary curative treatment modalities. In laparoscopic total or proximal gastrectomy with digestive tract reconstruction, the Roux-en-Y method or gastric tube reconstruction are favored for its simplicity, small anastomosis size, and its efficacy in preventing reflux esophagitis (5, 6). At present, esophagojejunostomy or esophagogastrostomy are the primary methods used for digestive tract reconstruction in total or proximal gastrectomy for patients with proximal gastric cancer (7, 8).

Esophagojejunal or esophagogastric anastomotic leakage stands as significant and potentially life-threatening complication following total or proximal gastrectomy for gastric cancer, with reported incidence rates between 2.3% and 13.6%, and the possibility of mortality reaching as high as 50% (9, 10). Among the multifactorial causes implicated in anastomotic leakage, inadequate anastomotic vascular perfusion has emerged as a pivotal factor affecting the healing of anastomoses (3, 11). However, assessing anastomotic vascular perfusion during total gastrectomy particular challenges. Typically, it relies on the surgeon’s visual judgment of subtle changes in the color or pulsation of small blood vessels within the digestive tract wall, which may fall short in providing a comprehensive assessment of the risk of (12, 13).

Indocyanine green fluorescence imaging (IGFI) has demonstrated its potential as an innovative surgical navigation technology for the precise localization of sentinel lymph nodes across various cancer types, including breast cancer and lung cancer (14). This technique is grounded in the direct visualization of the fluorescence emitted by indocyanine green (ICG) upon intravenous administration and exposure to near-infrared (NIR) light (15). Additionally, there is a growing body of literature highlighting the expanding utility of ICG in organ reconstruction and the evaluation of vascular perfusion at gastrointestinal anastomoses (16).

**Figure 1:**
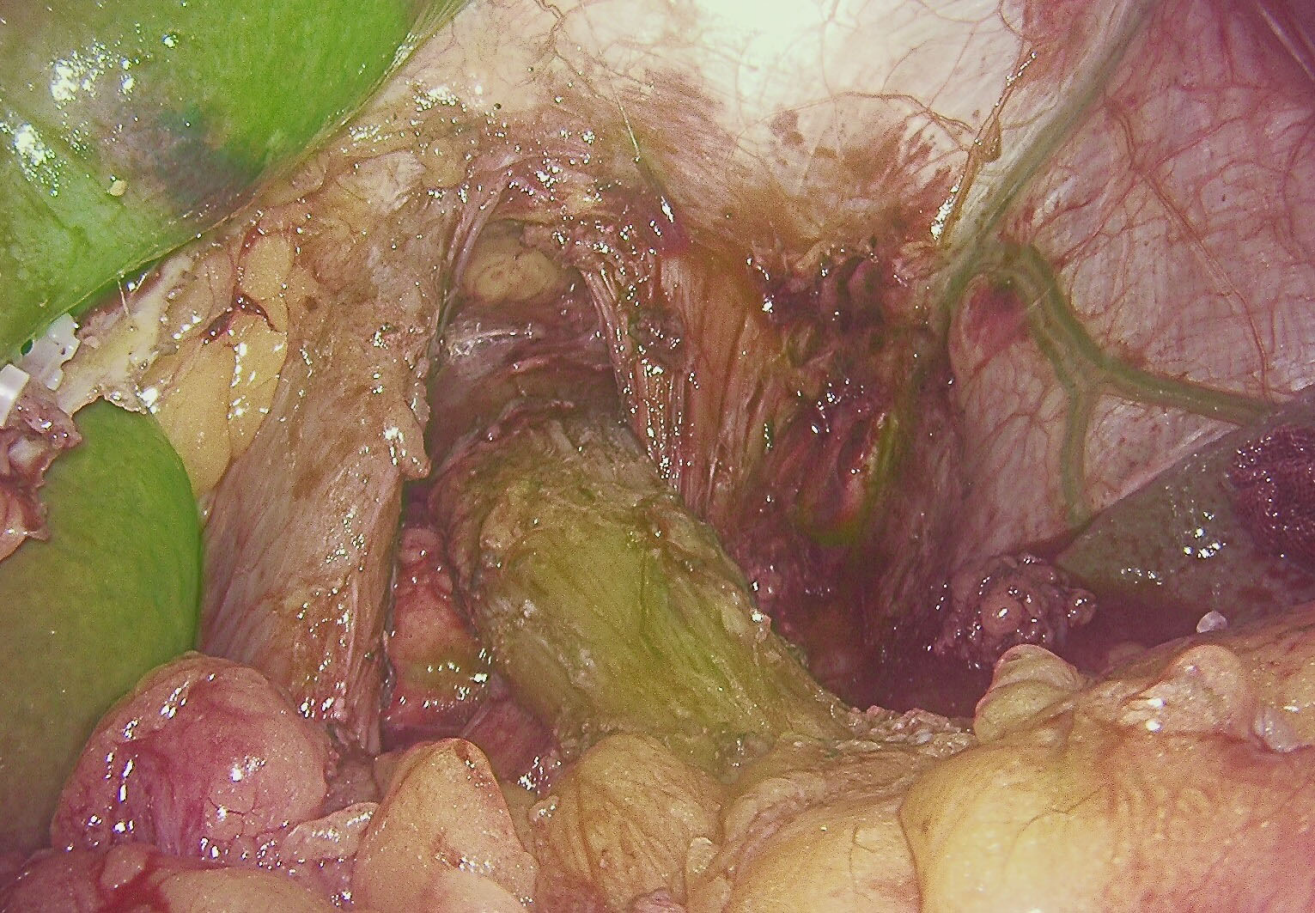
IGFI reveals satisfactory blood supply in the esophageal part before esophageal dissection.

Hence, the principal aim of this study is to present our findings regarding the efficacy of IGFI in assessing anastomotic blood supply during total or proximal gastrectomy for patients with gastric cancer. Our objective is to compare the results of this innovative approach with those of conventional total or proximal gastrectomy surgery, to explore the potential advantages of IGFI in enhancing patient outcome and care for gastric cancer treatment.

**Figure 2:**
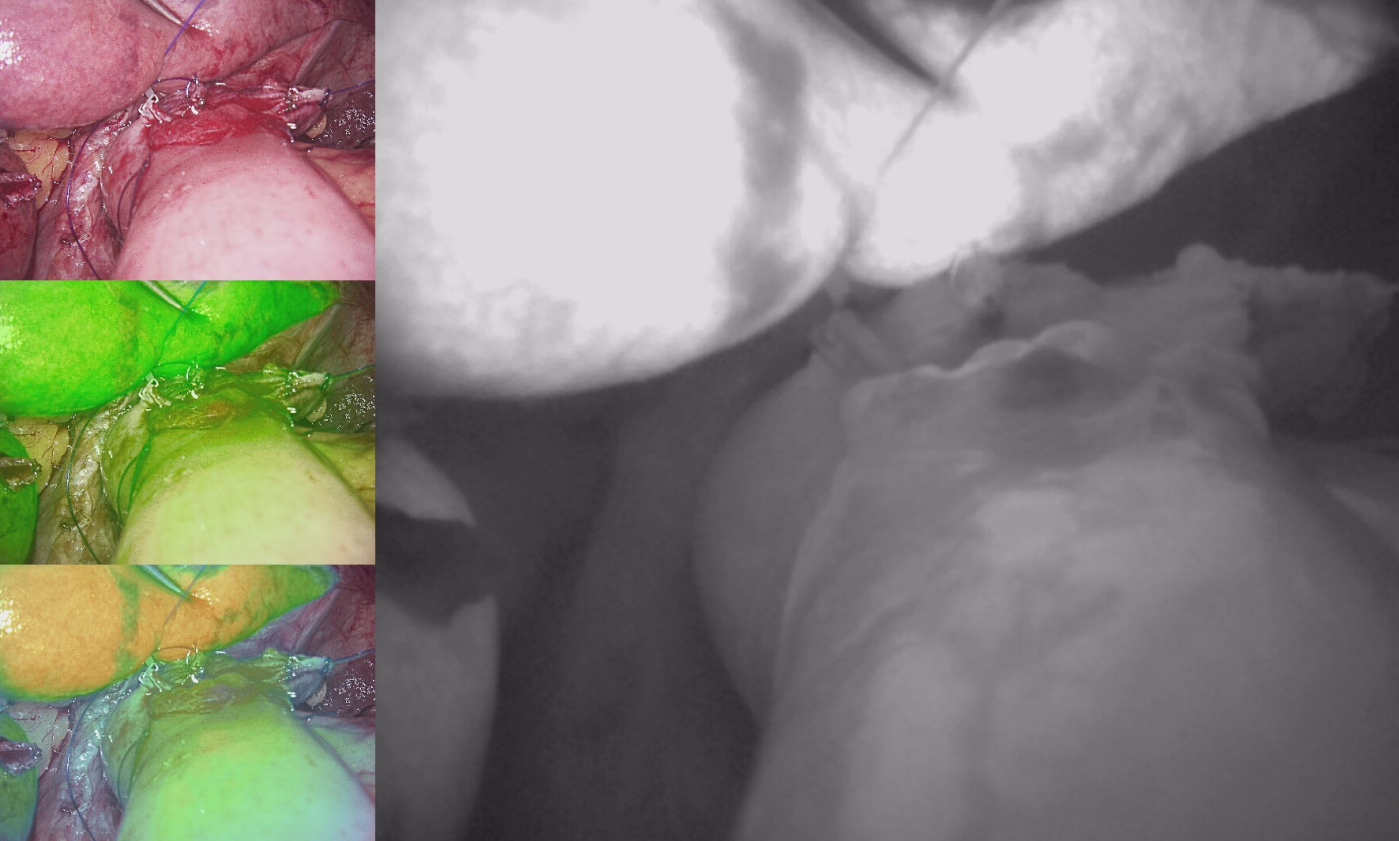
IGFI reveals satisfactory blood supply at the anastomotic site after Esophagojejunostomy.

## 2 Materials and methods

### 2.1 Study population and grouping

The ethics committee of the Peking Union Medical College/National Cancer Center/Chinese Academy of Medical Sciences granted approval for this study, and it adhered to the ethical standards outlined in the Declaration of Helsinki by the World Medical Association. Consecutive patients diagnosed with gastric adenocarcinoma and undergoing total or proximal gastrectomy were retrospectively enrolled from January 2019 to October 2021. The inclusion criteria for the study included patients aged 18 to 85 years, with preoperative gastroscopic pathology confirmed gastric adenocarcinoma, who underwent laparoscopic total or proximal gastrectomy with D2 lymph node dissection, and intraoperatively completed esophagojejunal Roux-en-Y anastomosis or esophagastric tube reconstruction. The exclusion criteria comprised unresectable conditions due to extensive local infiltration or distant metastasis; open surgeries or conversions to open surgeries during laparoscopic procedures; involving concomitant resection of other organs; preoperative administration of internal treatments, including chemotherapy, radiotherapy, or immunotherapy; remnant gastric cancer; and allergic reactions to ICG or iodides. Patients meeting the inclusion and exclusion criteria were divided into two groups: the IGFI group (86 patients) who underwent total or proximal gastrectomy with the use of indocyanine green fluorescence imaging, and the control group (92 patients) who received conventional laparoscopic total gastrectomy. The study adhered to the principles outlined in the Declaration of Helsinki. Detailed explanations of the procedures were provided to all patients before surgery, and written informed consent was obtained from each participant.

### 2.2 Surgical procedures

The surgical procedures in both groups were consistent with prior report (17), with all procedures conducted under laparoscopic direct visualization, encompassing total or proximal gastrectomy and D2 lymph node dissection. Every patient underwent esophagojejunal Roux-en-Y anastomosis or esophagastric tube reconstruction.

ICG has relatively low toxicity and has been approved by the United States Food and Drug Administration (FDA) and the European Medicines Agency (EMA). It is widely used worldwide. Its half-life in the blood is approximately 4 minutes, and it is metabolized by the liver and excreted into the bile duct, with no renal toxicity. The maximum tolerated dose per day is 2 mg/kg, and the recommended fluorescent dye dose per administration is 1.25 to 5.0 mg. Its emission of near-infrared light is an optical reaction and involves no radiation. These characteristics make ICG have extensive potential applications in medical imaging and related fields. A solution of ICG (Eisai, Tokyo, JP) at a concentration of 2.5 mg/mL was prepared. Before the gastrointestinal reconstruction process, it was administered intravenously via the peripheral vein. Initially, a 2 mL ICG solution was rapidly injected before the fluorescence observation (opto-cam 2100, Optomedic, Guangdong, China). The method for assessing the blood supply at the anastomotic site entailed the subsequent steps: after ICG injection, the laparoscope was switched to NIR fluorescence mode. Within 3-5 minutes, the green fluorescence emanating from the anastomotic site and the residual duodenum. Upon the appearance of green fluorescence, the modes were interchanged continuously between green and red-blue, facilitating the monitoring of the fluorescence intensity.

Considering the hepatic metabolism of ICG within 3-8 minutes following intravenous administration, the assessment of blood supply at the anastomotic site, residual end had to be concluded within 15 minutes post-injection. The evaluation of the blood supply at the anastomotic site was executed based on the fluorescence status and the Sherwinter scoring system as documented by Sherwinter et al. (18). The Sherwinter scoring system operates as follows: within 15 minutes of injection, under the NIR fluorescence mode: (1) Score 1: No fluorescence observed in the tissue; (2) Score 2: Patchy or intermittent areas of fluorescence observed; (3) Score 3: Uniform and complete fluorescence observed throughout the tissue; (4) Score 4: Complete fluorescence observed, with localized areas displaying high fluorescence intensity; (5) Score 5: Complete fluorescence observed, with a widespread and intense fluorescence throughout the tissue. For patients with a score below 3, the surgical resection was continued until uniform and complete fluorescence was achieved. After the completion of the digestive tract reconstruction, an additional 2 ml of ICG was administered, enabling a secondary assessment of perfusion. In the control group, the same anastomotic procedure was performed without the use of ICG, and the evaluation of anastomotic perfusion was conducted visually by the operating surgeon. In our evaluation of postoperative complications, we employed the Clavien-Dindo classification system—a widely accepted framework for grading the severity of complications (19).

### 2.3 Statistical analysis

Statistical analysis was conducted using SPSS software v24.0 (IBM, Armonk, NY, USA) and GraphPad Prism (version 8, GraphPad Prism Software Inc.). Normally distributed measurement data, presented as Mean ± SD, underwent analysis using t-tests or analysis of variance. For analysis of categorical data, frequencies and percentages were presented, and the Chi-squared test or Fisher’s exact test was employed. The Mann-Whitney test was utilized for ranked and non-normally distributed quantitative data. Univariate and multivariate logistic regression analyses were employed to assess factors associated with postoperative anastomotic leakage. In the univariate analysis, potential influencing factors related to anastomotic leakage were examined. Subsequently, the multivariate analysis further scrutinized the predictive role of each factor in postoperative anastomotic leakage, calculating 95% confidence intervals (CIs) for each risk factor. Statistical significance was considered at P < 0.05.

## 3 Results

### 3.1 Patient characteristics

In total, 178 patients met the predefined inclusion and exclusion criteria. The patient cohort exhibited a mean age of 51.3 ± 6.3 years, with males constituting 55.1% of the population. Among these patients, 86 were assigned to the IGFI group, while the remaining 92 comprised the control group. Both patient groups demonstrated equitable distribution in terms of age, sex, ASA score, BMI, anastomosis method, tumor grade, pathological T stage, pathological N stage, perineural invasion, and tumor size (**Table 1**).

**Table 1:** Baseline characteristics of gastric cancer patients between January, 2019 and October, 2021.

Characteristics	All patients	IGFI group	Control group	*P-*value
	(n = 178)	(n = 86)	(n = 92)	
Age at diagnosis, year, (n%)	51.3 ± 6.3	50.1 ± 7.2	52.3 ± 8.5	0.822
21-60	96 (53.9%)	45 (52.3%)	51 (55.4%)	
60-79	82 (46.1%)	41 (47.7%)	41 (44.6%)	
Sex, n (%)				0.562
Female	80 (44.9%)	34 (40.6%)	46 (50.0%)	
Male	98 (55.1%)	52 (59.4%)	46 (50.0%)	
ASA score				0.848
I	55 (30.8%)	27 (31.4%)	28 (30.4%)	
II	112 (62.9%)	52 (60.5%)	60 (65.2%)	
III	11(6.2%)	7 (8.1%)	4 (4.4%)	
BMI, kg/m2 (mean ± SD)	23.6 ± 2.5	22.4 ± 2.4	25.2 ± 1.8	0.334
Anastomosis method, (n%)				0.239
Esophagojejunostomy	92 (51.7%)	48 (55.8%)	44 (47.8%)	
Esophagogastrostomy	86 (48.3%)	38 (44.2%)	48 (52.2%)	
Tumor grade, (n%)				0.638
Poor or moderately	114 (64.0%)	58 (67.4%)	56 (60.9%)	
Mucinous or signet cell	64 (36.0%)	28 (32.6%)	36 (39.1%)	
Pathological T staging, (n%)				0.241
T1 or T2	82 (46.1%)	37 (44.2%)	45 (48.9%)	
T3 or T4	96 (53.9%)	49 (55.8%)	47 (51.1%)	
Pathological N staging, (n%)				0.836
N0	15 (8.4%)	9 (10.5%)	6 (6.5%)	
N1	63 (35.4%)	30 (34.9%)	33 (35.9%)	
N2	79 (44.4%)	39 (45.3%)	40 (43.5%)	
N3	21 (11.8%)	8 (9.3%)	13 (14.1%)	
Perineural invasion, (n%)				0.128
Yes	122 (68.5%)	53 (61.6%)	69 (75.0%)	
No	56 (31.5%)	33 (38.4%)	23 (25.0%)	
Tumor size, cm, (n%)				0.527
0 - 5	91 (51.1%)	50 (58.1%)	41 (44.6%)	
> 5	87 (48.9%)	36 (41.9%)	51 (55.4%)	

The incidence of conversion to open surgery was 5.0% (4 patients) in the IGFI group and 5.4% (5 patients) in the control group, displaying no statistically significant difference (p = 0.429). In the IGFI group, two patients underwent laparoscopic conversion to open surgery due to intraoperative bleeding, and two patients due to severe adhesions. In the control group, three patients underwent conversion to open surgery due to intraoperative bleeding, one patient due to severe adhesions, and one patient underwent open surgery due to the intraoperative identification of enlarged lymph nodes and vascular fusion. Regarding to the intraoperative adverse effects, it was observed that 2 patients in IGFI group and 3 patients in the control group encountered intraoperative bleeding. We timely converted the laparoscopic surgery to open surgery, performed hemostasis, and transfused red blood cells during the surgery. No additional intraoperative adverse reactions were reported. Estimates of blood loss did not significantly differ between the IGFI group (81.4 ± 25.6 mL) and the control group (82.6 ± 24.8 mL, p = 0.249), nor did the duration of postoperative hospital stays (7.1 ± 1.2 days for the IGFI group and 7.2 ± 0.8 days for the control group, p = 0.845). Operative time was assessed in minutes, in the IGFI group, the mean operative time was 172 minutes, with a standard deviation of 42.2. The control group demonstrated a mean operative time of 180 minutes, with a standard deviation of 40.1. The t-test statistical analysis revealed a p-value of 0.098, suggesting the absence of a statistically significant difference in operative time between the two groups. Regarding postoperative complications, the IGFI group exhibited a notably lower incidence of anastomotic leakage (2.3% vs. 8.7%, p = 0.046). There were no significant differences between the two groups concerning other postoperative complications, including bowel obstruction, surgical wound infection, bleeding, delayed gastric emptying, lung infection, and fever (all p > 0.05). Additionally, there were no reported cases of 30-day postoperative mortality in either group. Over the 6-month follow-up period, one patient in each group experienced peritoneal recurrence (**Table 2**).

**Table 2:** Perioperative data of patients.

Characteristics	IGFI group	Control group	*P-*value
	(n = 86)	(n = 92)	
Conversion to open, n (%)	4 (5.0%)	5 (5.4%)	0.429
Estimated blood loss in mL, mean ± SD	81.4 ± 25.6	82.6 ± 24.8	0.249
Operation time in min, mean ± SD	172 ± 42.2	180 ± 40.1	0.098
Hospital stay after operation (d, mean ± SD)	7.1 ± 1.2	7.2 ± 0.8	0.845
30 d post-operative mortality, n (%)	0	0	1
Time to first flatus, day (mean ± SD)	2.3 ± 1.4	2.5 ± 1.7	0.647
Time to Regular diet, day (mean ± SD)	5.5 ± 2.1	5.3 ± 2.5	0.251
Postoperative complications (Clavien-Dindo grades II, III)			
Pleural Effusion	2(2.3%)	0	0.905
Anastomotic Leakage	2 (2.3%)	8 (8.7%)	0.046
Bowel Obstruction	3 (3.5%)	4 (4.3%)	0.493
Surgical Wound Infection	5 (5.8%)	5 (5.4%)	0.242
Bleeding	2 (2.3%)	1 (1.1%)	0.332
Delayed gastric emptying	10 (12.0%)	14 (15.2%)	0.549
Lung infection	1 (1.2%)	0	0.452
Fever	7 (8.1%)	8 (8.7%)	0.735
Peritoneal recurrence	1 (1.2%)	1 (1.1%)	0.94

We performed multivariable logistic regression analysis to identify factors influencing anastomotic leakage. The findings underscored that advanced age beyond 60 years (OR = 2.589, 95% CI 1.731-2.869, p = 0.026) and diminished preoperative albumin levels (OR = 1.419, 95% CI 1.137-1.722, p = 0.022) were significantly associated with the occurrence of postoperative complications. Furthermore, treatment modality exhibited a notable correlation with anastomotic leakage, with the control group presenting a heightened risk compared to the IGFI group (OR = 1.633, 95% CI 1.319-1.885, p = 0.013) (**Table 3**).

**Table 3:** Multivariable analysis of factors influencing anastomotic leakage.

Characteristics	OR (95% CI)	*P* value
Age at diagnosis, year		
21-60	1 [Reference]	
60-79	2.589 (1.731-2.869)	0.026
Sex		
Female	1 [Reference]	
Male	1.186 (0.942-1.507)	0.644
ASA score		
I	1 [Reference]	
II	0.889 (0.551-1.245)	0.675
III	1.327 (0.865-1.632)	0.145
Anastomosis method		
Esophagojejunostomy	1 [Reference]	
Esophagogastrostomy	0.508 (0.477-1.224)	0.082
Tumor grade		
Poor or moderately	1 [Reference]	
Mucinous or signet cell	1.125 (0.877-1.399)	0.083
Pathological N staging		
N0	1 [Reference]	
N1/N2/N3	1.238 (1.093-1.371)	0.167
Treatment modality		
IGFI group	1 [Reference]	
Control group	1.633 (1.319-1.885)	0.013
Preoperative albumin level		
Nomal or above	1 [Reference]	
Below normal	1.419 (1.137-1.722)	0.022
Preoperative hemoglobin level		
Nomal or above	1 [Reference]	
Below normal	0.913 (0.708-1.255)	0.121

## 4 Discussion

The findings from our study highlight the potential utility of IGFI in the context of assessing anastomotic blood supply during surgical procedures for gastric cancer, specifically esophagojejunostomy or esophagogastrostomy (**Figures 1**, **2**). The results underscored several significant points that contribute to the ongoing discourse in the field.

The lower incidence of anastomotic leakage in the IGFI group underscores the potential clinical impact of this technology. During the course of the study, we observed that in six patients from the IGFI group, there was an intraoperative adjustment of the resection height prompted by insufficient perfusion, as indicated by IGFI. This dynamic response to real-time perfusion feedback highlights the adaptability afforded by IGFI in guiding surgical decisions. The rationale behind these adjustments was to optimize perfusion at the anastomotic site, potentially contributing to the subsequently observed lower anastomotic leakage rate in the IGFI group. Anastomotic leakage, a well-documented and severe postoperative complication, significantly impacts patient outcomes, elevating the risks of morbidity and mortality (6, 20). Adequate tissue perfusion stands as a pivotal factor for successful gastrointestinal tract anastomosis. Literature has confirmed the effectiveness of fluorescence-guided perfusion control, highlighting the significant value of this technology in reducing the risk of anastomotic leakage (21, 22). Through visual quantitative evaluation, it notably decreases the occurrence rate of postoperative anastomotic leakage attributed to impaired blood supply during surgery. The ability of IGFI to aid in the assessment of anastomotic blood supply, potentially reducing the likelihood of this complication, highlights its role in enhancing patient safety and improving surgical success rates (23, 24). Our findings suggest that real-time IGFI can effectively evaluate the blood supply at the resection margin during gastric cancer surgery. We recommend assessing blood flow pre-anastomosis, performing the corresponding anastomosis when satisfactory, and subsequently reassessing blood flow at the anastomotic site using IGFI. ICG rapidly diffuses from the blood vessels to the target anastomotic areas, and the fluorescence imaging remains stable for several minutes, with fluorescence gradually decreasing within 10 to 15 minutes. If necessary, a repeat ICG injection can be administered at this stage. In the IGFI group, anastomotic leakage occurred in two patients, compared to eight in the control group, with a statistically significant difference. However, the average hospital stay did not show a significant statistical difference. An in-depth review classified anastomotic leakage severity using the Clavien Dindo grading system. In the IGFI group, one patient had Grade A, and one had Grade B leakage, while in the control group, six had Grade A, one had Grade B, and one had Grade C leakage. Notably, Grade A cases in the IGFI group received timely discharge and oral antibiotic therapy, contributing to the overall comparable average hospital stay.

In our study, we found that older age and low preoperative albumin levels were independent risk factors for anastomotic leakage after esophagectomy. These findings are consistent with those of previous studies (25–27). However, some studies (28) have reported that age is not a risk factor for anastomotic leakage or that older age may actually reduce the risk. These conflicting findings may be due to a number of factors, such as differences in study populations, surgical techniques, and definitions of anastomotic leakage. Further research is needed to clarify the role of age in anastomotic leakage after gastric cancer surgery.

The observed lack of a statistically significant difference in operative time between the two groups (p=0.098) necessitates further investigation with a larger sample size to enhance the power of the analysis. Employing propensity score matching, along with a multivariable regression analysis that integrates recognized confounders, may offer a promising result to illuminate the genuine relationship between group assignment and operative time.

The results of our study have the potential to have a significant impact on current surgical practice for gastric cancer. First, our findings suggest that IGFI is a feasible and safe tool for intraoperative assessment of anastomotic blood supply. IGFI could be used to identify patients with suboptimal anastomotic blood supply. This information could then be used to make intraoperative adjustments to the anastomosis, such as selecting a different resection point. Also, our findings also suggest that IGFI may be associated with a lower incidence of postoperative anastomotic leakage. This is a promising finding, as it suggests that IGFI could be used to reduce the risk of this serious complication.

Despite the notable findings, our study has several limitations that should be considered. The retrospective nature of the study design inherently limits the ability to establish causal relationships, and the lack of propensity score matching in our study could potentially introduce bias and affect the robustness of our findings. The relatively limited sample size might have affected the statistical power and precision of the study, warranting caution in interpreting the findings. Additionally, the absence of long-term follow-up data restricts the ability to assess the enduring impact of IGFI on patient outcomes beyond the immediate postoperative period. Future studies, especially prospective, randomized trials with larger sample sizes and longer follow-up durations are necessary to address these limitations and further elucidate the clinical implications of IGFI in the context of gastric cancer surgeries.

In conclusion, our study supports the integration of IGFI as a valuable intraoperative tool for assessing anastomotic blood supply during esophagojejunostomy or esophagogastrostomy for gastric cancer (29). The findings emphasize the potential of IGFI to contribute to more efficient surgical procedures, reduced postoperative complications, and improved patient outcomes. Further research and clinical validation are necessary to fully elucidate the benefits of IGFI and its role in enhancing the standard of care for patients undergoing gastric cancer surgeries.

## Data availability statement

The original contributions presented in the study are included in the article/supplementary material. Further inquiries can be directed to the corresponding authors.

## Ethics statement

The studies involving humans were approved by National Cancer Center, Chinese Academy of Medical Sciences. The studies were conducted in accordance with the local legislation and institutional requirements. Written informed consent for participation was not required from the participants or the participants’ legal guardians/next of kin in accordance with the national legislation and institutional requirements.

## Author contributions

PW: Conceptualization, Data curation, Formal analysis, Writing – original draft. YT: Data curation, Formal analysis, Investigation, Project administration, Validation, Writing – original draft. YD: Data curation, Investigation, Software, Validation, Writing – review & editing. YZ: Conceptualization, Funding acquisition, Methodology, Writing – review & editing.
